# Perforated gastric cancer: a critical appraisal

**DOI:** 10.1007/s12672-021-00410-z

**Published:** 2021-05-15

**Authors:** Sara Di Carlo, Marzia Franceschilli, Piero Rossi, Giuseppe Cavallaro, Maurizio Cardi, Danilo Vinci, Simone Sibio

**Affiliations:** 1 Department of Surgery, Minimally Invasive Unit, University of Rome “Tor Vergata”, Rome, Italy; 2 Department of Surgical Sciences, University of Rome “Tor Vergata”, Minimally Invasive Unit, Tor Vergata Hospital, Rome, Italy; 3 Department of Surgery Pietro Valdoni, Unit of Oncologic and Minimally Invasive Surgery, Sapienza University of Rome, Viale del Policlinico 155, 00161 Rome, Italy

**Keywords:** Gastric cancer, Perforated gastric tumor, Perforation, Peritonitis, Gastrectomy

## Abstract

Gastric cancer perforation is a life-threatening condition that accounts for less than 5% of all gastric cancer patients and typically requires emergency surgery. However, preoperative diagnosis is difficult and management has a dual purpose: to treat peritonitis and to achieve a curative resection. The optimal surgical strategy is still unclear and prognosis remains poor. A search of the literature was performed using MEDLINE databases (Pubmed, EMBASE, Web of Science and Cochrane) using terms such as “perforated gastric cancer”, “perforated gastric cancer and surgery”, “perforated gastric tumour” and “gastric cancer perforated”. Case reports, other reviews, non-english written papers and papers written before 2010 were excluded. Eight articles published between 2010 and 2020 matched the inclusion criteria for this review. Perforated gastric cancer was more prevalent in elderly males. Distal stomach was most frequently involved. Preoperative diagnosis was uncommon. Mortality rates ranged from 2 to 46%. Patients able to receive an R0 resection demonstrated better long-term survival compared with patients who had simple closure procedures. Laparoscopic procedure was mentioned only in one study. In an emergency situation, curative RO resection should always be offered in patients without multiple adverse factors. A surgical strategy using laparoscopic local repair as first step of surgery to resolve the peritonitis followed by a radical open or laparoscopic gastrectomy with lymphadenectomy could be considered. A balance between emergency and oncological needs should drive the surgical choice on a case by case basis.

## Introduction

Gastric cancer (GC) is one of the leading causes of death worldwide although its incidence has been declined [1, 2]. The spontaneous perforation of gastric cancer (PGC) is a life-threatening condition that occurs in less than 5% of all gastric cancer patients and it is a surgical emergency fraught with numerous challenges. In most circumstances, PGC is not Known preoperatively and is associated to advanced disease stage [3]. It is difficult to preoperatively diagnose PGC because its symptoms are the same as those of a perforated gastric ulcer. Patients experiencing gastric perforation exhibit acute onset abdominal pain with evidence of free air on plain abdominal X-ray. Furthermore, it may be difficult to distinguish a gastric cancer from a gastric ulcer at the time of surgery unless a pre-operative diagnosis of GC is known or a clear metastatic disease is found at laparotomy. Therefore, diagnosis of malignancy is made post-operatively in most of the cases [4, 5]. An intraoperative frozen section could help in the matter. When diffuse peritonitis is diagnosed, emergent surgery is necessary. The aims of surgery in these patients are two-fold: to resolve peritonitis and to achieve a curative resection. The ideal option in treating the malignancy is unclear and it may be a result of various factors such as those related to the emergency presentation (hemodynamic stability of the patient, extent of peritonitis, active comorbidities) and those depending on the surgical expertise and the stage of the malignancy [6, 7]. The surgical procedures range from a simple lavage and perforation closure to resection that can be performed by employing the one-stage or two stage technique. The first procedure treats life-threatening peritonitis followed by the second procedure which includes definitive gastrectomy with appropriate lymphadenectomy [4, 5]. The short-term outcome in these patients is often poor due to the septic complications following the peritonitis and to the post-operative morbidity. Moreover, also the long-term outcome may be poor due to the advanced stage of cancer and the early development of peritoneal metastases related to the perforation [8].

The optimal surgical strategy for PGC is still debated. This paper aims to review the surgical options in case of perforated gastric cancer and focuses on surgical outcomes, survival rates and pre-operative diagnosis.

### Search strategy

An electronic search was conducted using MEDLINE databases (Pubmed, EMBASE, Cochrane and Web of Science) matching terms such as “perforated gastric cancer”, “perforated gastric cancer and surgery”, “perforated gastric tumor” and “gastric cancer perforated”. All publications in English between 2010 and 2020 were reviewed. Case reports, reviews, meta-analyses, abstracts, non-english papers and letters written before 2010 were excluded. Cases describing iatrogenic gastric perforation related to endoscopic mucosal resection (EMR) or endoscopic submucosal resection (ESD) for early gastric cancer were not included. Pathological gastric perforations caused by gastric lymphoma and metastatic melanoma or lung cancer were not considered. The following data were retrieved from the publications: author, publication year, country, number of patients, gender, age, tumor location, tumor stage, pre-operative diagnosis of gastric cancer, type of emergency surgery (repair versus resection), type of gastrectomy (one-stage versus two stage), use of laparoscopic procedure, R0 resections, post-operative morbidity and mortality and survival time. A statistical analysis comparing survival curves for each surgical procedure have been conducted by mean of log rank test; a *p*-value < 0.05 has been considered significant.

### Patients characteristics

Eight studies published between 2010 and 2020 matched the inclusion criteria in this review. The preoperative characteristics of the patient population are shown in Table 1. There is a great heterogeneity in patients characteristics due to the wide difference in the consistency of sample which range from 8 to 2964 patients. Patients age ranged from 60 to 79 years (mean 70 year) and they were predominantly males. Data on preoperative diagnosis of gastric cancer were reported only in five studies, and account for a mean of 38% of the patients [5, 7, 9–11]. The most common tumor location was the distal part of the stomach in 37% of the patients, followed by the middle third in 36% and the upper third in 27%. The majority of the patients (66%) presented stage III or IV disease.

**Table 1:** Preoperative Characteristics of the patient population

Study	Year	Country	No of patients	Median age	Males (%)	Preop diagnosis (%)	Stage (%)	
							III	IV
Tsujimoto et al. [13]	2010	Japan	8	64.5	65.5	51.7	75	12
Tan et al. [7]	2011	Singapore	9	76	56	22	73	27
Kim et al. [11]	2014	South Korea	35	NR	65.7	NR	60	NR
Hata et al. [12]	2014	Japan	514	NR	74.6	NR	55	34
Ignjatovic et al. [10]	2016	Serbia	11	60	72.8	18	73	27
Wang et al. [9]	2017	China	29	77	65.5	51.7	55	34
Fisher et al. [15]	2020	Danville	2964	79	59.4	NR	36	23
Kim et al. [5]	2020	Korea	43	69	55	42	75	25

### Surgical procedures and outcomes

**Fig. 1:**
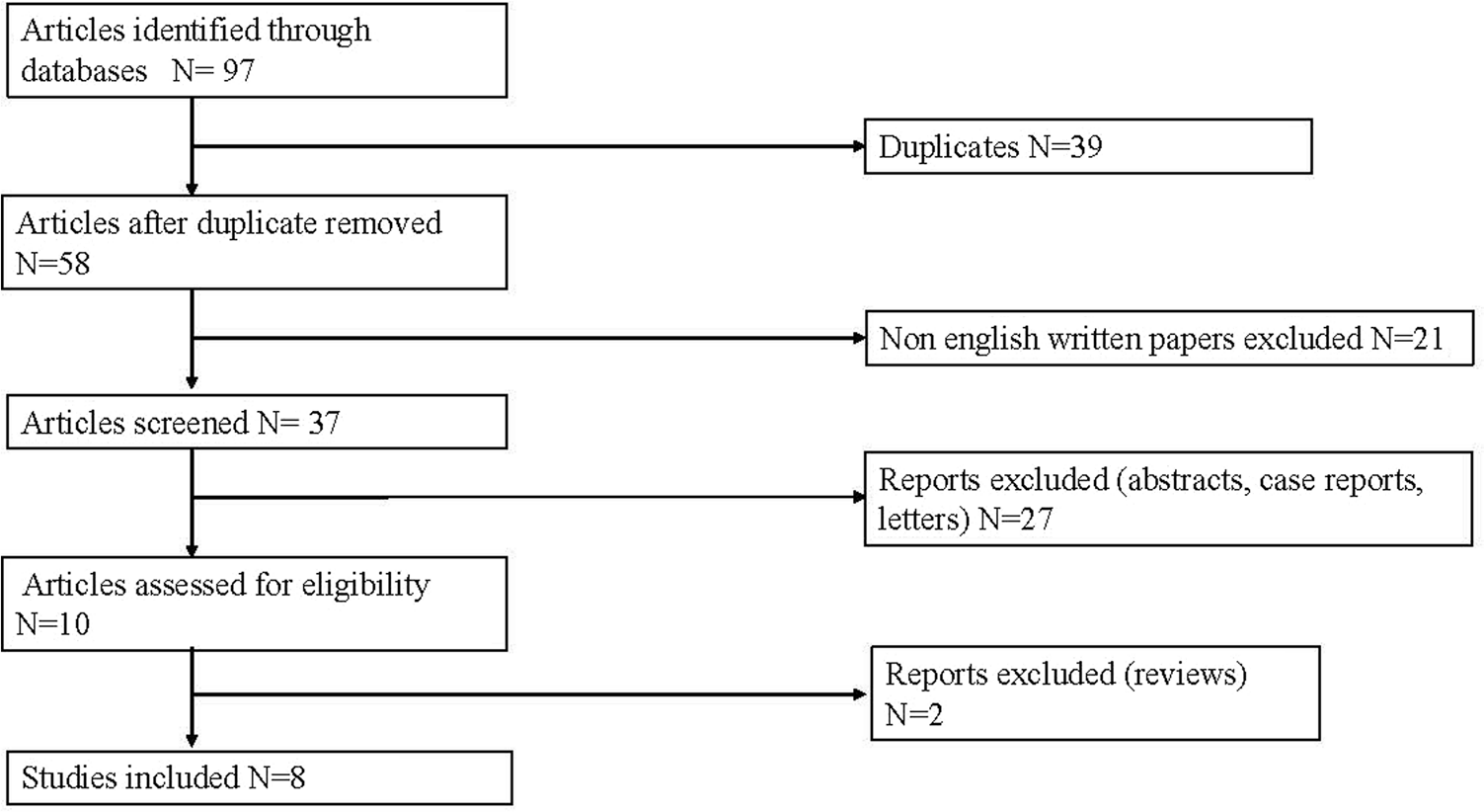
PRISMA diagram for articles include in the review

**Fig. 2:**
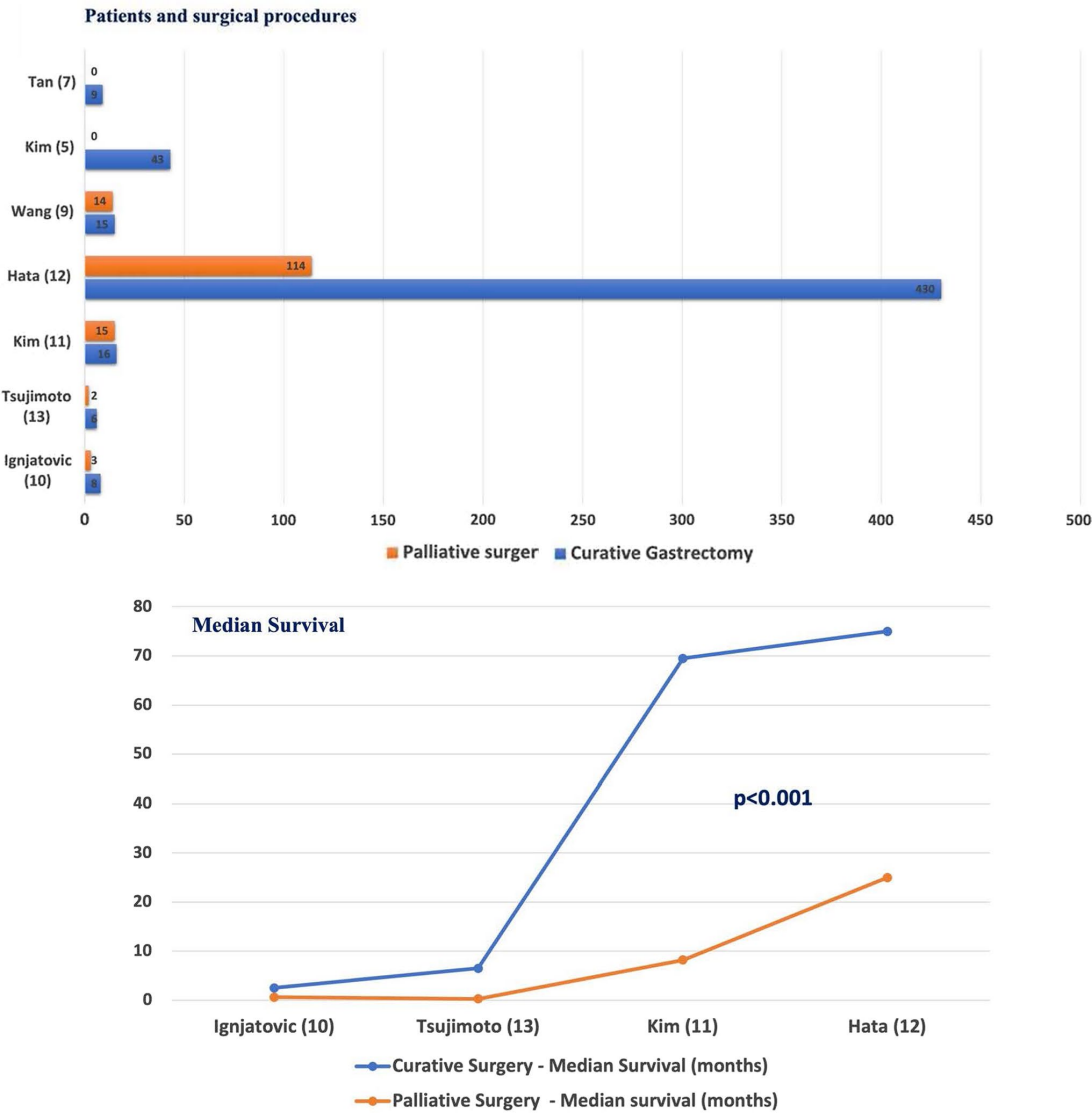
Surgical procedures and survival

A total of 476 patients underwent total or subtotal gastrectomy as a one-stage or staged operation according to their general conditions. Only one study mentioned a laparoscopic approach in 38 patients who underwent laparoscopic gastrectomy performed by an experienced surgeon [5]. Moreover, 140 patients underwent repair with simple closure or omental patch and 12 patients were treated non-surgically due to poor conditions or refusal. 441 patients had single-stage gastrectomy and 55 had only damage-control surgery or an initial conservative management followed by gastrectomy (Table 2). Poor overall general conditions, high clinical risk or advanced disease were reasons provided for simple closure. Two-stage operations for improved oncological outcome were discussed in three studies [10–12]. Wang et al. [9] described a scheduled gastrectomy following a primary closure in 4 patients according to clinical conditions. Hata et al. [12] reported their experience on 514 patients. Of them, 388 patients underwent gastrectomy, completed as one stage in 376 and in two-stage in the remnant 12. Of the 114 cases treated by performing simple closure or omental patch, 38 received a second surgery: gastrectomy was performed in 32 but not in the remaining 6 cases because of advanced stage (Fig. 1). 12 more patients were treated conservatively due to limited peritonitis and subsequently 10 of them received gastrectomy and were included in the group of patients who underwent two stage procedure. Ignjatovic et al. [10], out of 11 patients treated, performed one-stage procedure in 3 (27.2%) and two-stage approach in 5 (45.6%). Simple closure with omental patch was performed in the other 3 patients. Post-operative morbidity was reported only in one study and accounted for 35% [5]. Overall operative mortality rates changed very widely throughout all the studies, ranging from 2 to 46% (mean 18%). As a common sense, usually surgeon skills did not influence the surgical choice. Indeed, the laparoscopic approach can be performed only in selected referral centers where an advanced expertise in laparoscopic gastric surgery is available. Data regarding number and type of surgical procedures are reported in Fig. 2.

**Table 2:** Management of patients with perforated cancer and surgical outcomes (patients number)

Study	Total or Subt. Gastrectomy	1-stage	2-stage	Omental patch	No surgery	R0(%)	30d-mortality(%)
Tsujimoto et al. [13]	6	NR	NR	1	1	NR	25
Tan et al. [7]	9	NR	NR	0	0	NR	NR
Kim et al. [11]	16	16	0	15	4	35	28
Hata et al. [12]	388	376	12	114	2	50–78	11–2
Ignjatovic et al. [10]	8	3	5	3	0	NR	46
Wang et al. [9]	15	NR	NR	7	7	52	14
Fisher et al. [15]	NR	NR	NR	NR	NR	82	6
Kim et al. (5)	43	43	0	0	0	75	15

### Prognostic factors and survival

Different survival outcomes were also reported among the studies. As expected, R0 resection was always associated to improved survival if compared to R1/2 or palliative procedures. Data regarding median survival for each procedure are shown in Fig. 2. Differences between survival curves showed statistical significance (*p* < 0.001—log-rank test). Kim et al. [5] performed radical gastrectomy with D2 lymphadenectomy in 35 patients and reported an overall 5-year survival of 19.5%. The mean survival time was 23 months ranging from 2 months for patients who underwent palliative surgery to 75 months for patients who underwent curative resection. Only three papers report data about adjuvant chemotherapy but none points out differences in survival between patients who underwent adjuvant chemotherapy and those who did not [5, 7, 15].

## Discussion

Perforated gastric cancer (PGC) accounts for less than 1% of cases of acute abdomen but it represents the main oncologic emergency after major bleeding [13]. Although gastric cancer is the third leading cause of death from cancer worldwide [14], the management of PGC is still debated due to the lack of clinical guidelines supporting a specific algorithm in an emergency situation where surgery has a dual purpose: treating life-threatening peritonitis and curing gastric cancer. Even if gastric cancer can be diagnosed preoperatively or intra-operatively, the choice about treatment in PGC depends on several factors regarding emergency, oncologic and patient variables, such as severity of peritonitis, hemodynamic instability, sepsis, presence of comorbidities, presence of metastases at exploration [8].

In this review, only 38% of the patients had a pre-operative diagnosis of gastric cancer and this is in line with data reported in other reviews on the topic [4, 8]. PGC is a rare condition that accounts for less than 5% of all gastric cancers [8, 10] but it is very difficult to diagnose before and at surgery and the treatment goal, balancing oncologic with emergency criteria, still represents a challenging issue. Given that patients with benign and malignant gastric perforation exhibit similar symptoms such as generalized abdominal pain, rebound tenderness, poor systemic conditions caused by peritonitis that make a detailed clinical examination difficult, the diagnosis of gastric cancer is frequently made post-operatively. Moreover, intraoperative endoscopy and frozen section are often not available in emergency; furthermore, inflammatory changes associated with peritonitis resemble those caused by tumor invasion leading to misinterpretation and overestimation of intraoperative findings [9, 13]. In this review, 66% of patients had stage III-IV disease, and the overall mortality ranged from 2 to 46%. In the past, primary closure was the surgical treatment of perforation because PGC was thought to indicate terminal disease. In some cases, leakage at the suture site required a second operation after which the patient’s general conditions worsened [7]. Since then, several reports on PGC have shown a significantly better prognosis for patients who underwent curative resection if compared to those who underwent non-curative resection [4, 5, 7, 9, 12]. Two surgical methods are used in management of PGC. Single stage radical D2 gastrectomy is recommended if the patient’s general conditions are favorable. Hata et al. [12], in their study, found a statistically significant difference in operative mortality between one-stage and two-stage gastrectomy patients (11.4% versus 1.9%). In the same series, a curative resection (R0) was achieved in 50% of the one-stage gastrectomy group and in 78.4% of the two-stage gastrectomy group. Regardless of whether patients underwent a one-stage or two-stage gastrectomy, curative R0 resection improved survival. This indicated that when curative R0 resection cannot be immediately performed due to peritonitis, palliative or non-curative gastrectomy should be avoided and two-stage gastrectomy should be planned following peritonitis recovery. In the same study, Hata et al. [12] described a median survival of 75 months and a 5-year survival rate of about 50% when a curative resection was possible. Similar results were obtained by Ignjatovic et al. [10] who demonstrated a higher survival rate among patients who underwent curative resection if compared to those who underwent simple closure with omental patch (75.77 days vs 18 days). Also the oncologic value of the surgical procedure seems to be worsened by the emergency setting of these patients: Fisher et al. [15] in their paper found that patients who underwent urgent surgery for gastric cancer had significantly worse quality resections with decreased lymph node retrieved and increased positive margins as well as increased 30 day and overall mortality if compared to patients undergoing elective surgery.

In regards to the recurrence rate after PGC, Tsujimoto et al. [13] showed that there were no significant differences between perforated and non-perforated gastric cancer. Mahar et al. [4] also showed that peritoneal contamination due to perforation did not affect survival as it was thought in the past. According to Tan et al. [7] long-term survival was dependent on the stage of malignancy. As demonstrated in his small series advanced stage of the gastric malignancy and the possibility of tumor seeding of the peritoneal cavity led to unfavorable outcomes. According to these results, we could conclude that emergency presentation, regardless to the severity of the clinical findings, would not affect survival except for its negative impact on quality of oncologic resection.

Nowadays, minimally invasive surgery can be considered a suitable option for perforated gastric or duodenal ulcer, both for diagnosis or elective resection [16–18]. Laparoscopic gastrectomy appeared comparable with open technique in terms of overall and disease-free survival [19, 20]. On the other hand, laparoscopic gastrectomy in emergency cannot be considered a standard choice so far. Kim et al. [5] reported a laparoscopic gastrectomy with D2 lymphadenectomy performed in 35 of 43 patients with PGC. In their high specialized experience primary laparoscopic gastrectomy was recommended in emergency only when an appropriate expertise is available. Indeed, laparoscopic primary gastrectomy with lymphadenectomy could be the most promising treatment for PGC if the patient general conditions and surgeon ability allow it but it cannot be still considered the standard of care.

In conclusion, the most important goal for PGC is to achieve curative R0 resection, regardless of whether the surgical approach is a one-stage or two-stage gastrectomy. When gastric cancer can be diagnosed before or at surgery, one-stage gastrectomy should be performed in those cases with limited peritonitis and when a curative R0 resection can be achieved. On the contrary, if curative R0 resection cannot be achieved due to diffuse peritonitis, it is important to treat the peritonitis first and then to plan a two-stage gastrectomy. Whether the procedure should be performed laparoscopically or open depends on the surgical expertise and on the patient’s general conditions. A laparoscopic approach to treat the peritonitis and the perforation followed by a curative surgery could definitely reduce intra-abdominal adhesions and facilitate the staged gastrectomy. Future studies should evaluate how to improve the preoperative diagnosis as well as the more appropriate surgical choice, also considering the promising results offered by neoadjuvant chemotherapy and the still debated indications of the intraoperative chemotherapy in order to reduce the cancer cell seeding and increase long-term survival. Also, the prognostic role of adjuvant treatments in this subset of gastric cancer patients should be better assessed: only three papers reported data about adjuvant chemotherapy delivery but none pointed out its role in conditioning survival and no statistical differences have been reported between patients undergoing it or not. Since gastric cancer has the highest rate of peritoneal metastasis, prevention of peritoneal carcinomatosis is a critical need. In selected cases of PGC, laparoscopic surgery associated with hyperthermic intraoperative chemotherapy (HIPEC) has shown some benefit in reducing the risk of peritoneal recurrence [21, 22] although the real mechanisms regulating peritoneal seeding of tumor cells from GI cancers still need to be better clarified [23–26].

As a general recommendation, we can conclude that the best results in terms of surgical outcome and overall survival are obtained when treatment strategy is tailored on general conditions of patients, especially in regards to the extent of peritonitis; a balance between oncologic and emergency criteria should be the guiding light for the treatment choice on a case-by-case basis. One stage or staged gastrectomy with curative intent (i.e. oncological criteria) should always be preferred if technically feasible when a gastric tumor is suspected even when a definitive histology is not provided. Simple closure or omental patch can be considered valuable options instead of gastrectomy when life-threatening conditions are prevalent; similar results in terms of oncologic outcome are reported for these patients if radical surgery is postponed and neoadjuvant and/or adjuvant chemotherapy are administered. Indications for laparoscopic surgery remain limited.

This study has some limitations due to the heterogeneity of the samples, the rarity of PGC, the lack of the histopathology among the studies, the lack of a homogeneous assessment of clinical conditions that have led to a surgical approach instead than another. Future studies should standardize a proper algorithm to deal with this life-threatening condition in order to ameliorate the overall survival of these patients.

## Data Availability

Data are available upon request.
